# Synthesis and Characterization of Indium Tin Oxide Nanowires with Surface Modification of Silver Nanoparticles by Electrochemical Method

**DOI:** 10.3390/nano12060897

**Published:** 2022-03-08

**Authors:** Shu-Meng Yang, Hsi-Kai Yen, Kuo-Chang Lu

**Affiliations:** 1 Department of Materials Science and Engineering, National Cheng Kung University, Tainan 701, Taiwan; n56074287@gs.ncku.edu.tw (S.-M.Y.); n56091302@gs.ncku.edu.tw (H.-K.Y.); 2 Core Facility Center, National Cheng Kung University, Tainan 701, Taiwan

**Keywords:** indium tin oxide, nanowires, chemical vapor deposition, electrochemical, nanoparticles

## Abstract

In this study, indium tin oxide nanowires (ITO NWs) with high density and crystallinity were synthesized by chemical vapor deposition (CVD) via a vapor–liquid–solid (VLS) route; the NWs were decorated with 1 at% and 3 at% silver nanoparticles on the surface by a unique electrochemical method. The ITO NWs possessed great morphologies with lengths of 5~10 μm and an average diameter of 58.1 nm. Characterization was conducted through transmission electron microscopy (TEM), energy dispersive spectroscopy (EDS), X-ray diffraction (XRD) and X-ray photoelectron spectroscope (XPS) to identify the structure and composition of the ITO NWs. The room temperature photoluminescence (PL) studies show that the ITO NWs were of visible light-emitting properties, and there were a large number of oxygen vacancies on the surface. The successful modification of Ag was confirmed by TEM, XRD and XPS. PL analysis reveals that there was an extra Ag signal at around 1.895 eV, indicating the potential application of Ag-ITO NWs as nanoscale optical materials. Electrical measurements show that more Ag nanoparticles on the surface of ITO NWs contributed to higher resistivity, demonstrating the change in the electron transmission channel of the Ag-ITO NWs. ITO NWs and Ag-ITO NWs are expected to enhance the performance of electronic and optoelectronic devices.

## 1. Introduction

With the development of nanotechnology, nanodevices are undergoing a huge upgrade over the past few years. To fulfill the requirements of this technology, studies have been conducted on diverse nanomaterials [1,2,3]. Among them, indium tin oxide (ITO) is a transparent conducting oxide (TCO) with excellent characteristics of high light transmission and low resistivity; thus, it is extensively used in optoelectronic products, including liquid crystal displays [4], solar cells [5], flat-panel displays [6], and light-emitting diodes [7]. With low resistance and abundant lattice defects on the surface, ITO can also be used as gas sensor materials [8,9]. Furthermore, similar to NiSi nanowires, ITO nanowires may also be used as interconnects in integrated nanoscale devices [10]. ITO thin films have been studied extensively by several researchers [11,12,13]; however, there have been relatively few reports on one-dimensional ITO NWs and even fewer studies on modified-ITO NWs.

In recent years, reports demonstrated the successful synthesis of ITO NWs with good performance via different techniques, including pulsed laser deposition (PLD) [14], carbothermal evaporation process [15], CVD [16], and the template method [17], among which, CVD is a better choice since it is more straightforward, economical and practical. On the other hand, there are some studies in the modification of nanostructured materials, such as decorating Ag nanoclusters on SnO_2_ NWs for ethanol gas sensing [18], Ge-doped ITO nanocomposites for photovoltaic application [19], and photochemical deposition of Pd onto TiO_2_ NWs for hydrogenation [20]. With strong absorption and scattering of light, large electrical conductivity, easy detectability in low concentration and amazing biocompatibility, silver nanoparticles have been applied in various optoelectronic devices, such as UV lasers, infrared detectors and piezoelectric transducers. For noble metals, the surface plasmon resonance effect is promising and may increase optical absorption and light transmission rate, enabling the potential of silver nanoparticles to extend the lifetime of electronic devices [21,22,23].

In this work, we report a unique two-step method to synthesize Ag nanoparticle-decorated ITO (Ag-ITO) NWs. In the first step, ITO NWs were synthesized by the CVD method via the VLS route. In the second step, Ag nanoparticles were decorated on the surface of ITO NWs by the electrochemical deposition method. Therefore, the amount of decorated Ag nanoparticles was decided by reaction time. We characterized the as-grown ITO and Ag-ITO NWs by SEM, TEM, XRD and XPS. Additionally, electrical resistivity and PL measurements of ITO and Ag-ITO NWs were performed to investigate how the Ag-decoration impacts the structure, electrical and optical properties of ITO for a better understanding of this interesting material.

## 2. Materials and Methods

### 2.1. Synthesis of ITO Nanowires

In this experiment, ITO NWs were synthesized in a horizontal tube furnace with three heating zones via a catalyst-assisted CVD-VLS process. Firstly, a mixture of indium oxide, tin oxide powder (Alfa Aesar, Ward Hill, MA, USA, purity 99.9%) and active charcoal (Sigma-Aldrich, St. Louis, MO, USA) was loaded into an alumina boat. Then, the alumina boat was placed at the center of the first heating zone. In another alumina boat, placed downstream outside the third heating zone of the furnace, are sonically cleaned Si substrates with a 10 nm-thick Au layer on top. The temperature of the first heating zone was increased to 900 ℃, while the temperature of the third heating zone was set at 865 ℃. Argon gas (Ar, purity 99.995%) with a flow rate of 100 sccm was applied as the carrier gas. After 60 min of reaction, the furnace was cooled down to room temperature.

### 2.2. Surface Modification of Ag Nanoparticles by Electrochemical Method

**Figure 1:**
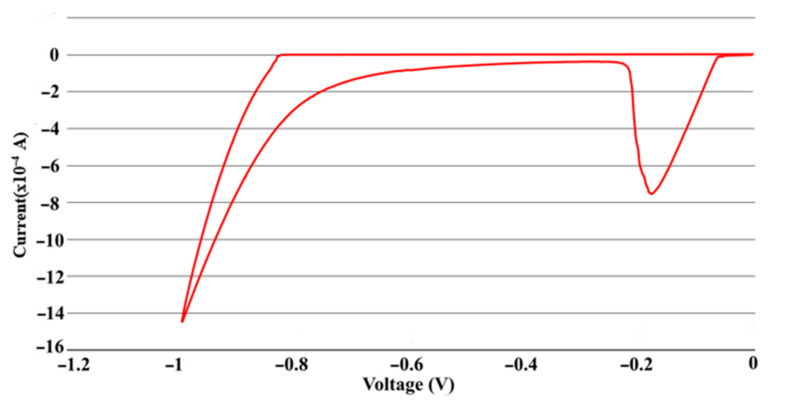
CV measurements on 0 to −1 V to acquire the reduced voltage of silver.

Following the synthesis of ITO NWs, Ag nanoparticles were decorated on their surface by the electrochemical method. In the two-electrode system, both the counter electrode (CE) and the reference electrode (RE) were platinum, while the working electrode (WE) is an as-grown ITO NWs substrate. The solution was selected to be mixed with AgNO_3_ and KNO_3_, with a ratio of 3:1. KNO_3_ enhanced Ag-decoration on the surface of the ITO nanowire due to the synergy effect [24]. Firstly, we placed the as-grown ITO NWs substrate at the position of WE, and the Pt sheet was placed at the position of CE/RE. Both of them were put into the prepared solution face-to-face. Then, the cyclic voltammetry (CV) method was used to find the voltage with which the reductive reaction would occur; the voltage applied was from 0 to −1 V and then back to the origin to obtain the reduction potential of silver ions, to be about −0.2 V with a scan rate of 50 mV. The half CV diagram is shown in Figure 1. At this potential, the silver ions in the solution could be reduced to silver nanoparticles on the surface of the ITO NWs, and then reacted by the amperometry method at a potential of −0.2 V for 50 s and 100 s to acquire 1 at% and 3 at% Ag-decorated ITO NWs. The relationship between time and the percentage of Ag deposits come from several experiments on an ITO nanowire by EDS to obtain the precise atomic percentage of silver nanoparticles decorated on an ITO nanowire.

### 2.3. Characterization and Property Measurements of ITO and Ag-ITO NWs

Characterization of the grown nanowires was conducted with FE-SEM (Hitachi SU8000, Tokyo, Japan), HR-TEM (JEOL JEM-2100F CS STEM, Tokyo, Japan), grazing angle XRD (Bruker D8 discover, Fitchburg, WI, USA), and XPS (PHI 5000 Versaprobe, ULVAC-PHI, Chigasaki, Japan). For the optical properties, PL (Horiba LabRam-HR, Longjumeau, France) measurements were conducted. For the electrical properties, we measured resistivity using a special method reported by Gu et al. [25]; this accurate method was designed for the resistivity of a single nanowire, eliminating possible errors, including contact resistances or Schottky contacts existing in two-probe measurements and four-probe measurements, respectively. To fabricate the nanodevice for the electrical measurements, the as-grown ITO NWs substrate was sonicated in deionized water for the separation of nanowires from the substrate; then droplets of solution containing ITO NWs were dripped on a previously prepared Si/SiO_2_ wafer with four independent Ag electrodes. Each nanowire was linked to four independent electrodes with Pt via selective deposition by a focused ion beam (FIB, FEI Nova-200 NanoLab Compatible, Hillsboro, OR, USA). The fabrication process is shown in Supplementary Materials Figure S2.

## 3. Results and Discussion

### 3.1. Synthesis and Characterization of ITO and Ag-ITO NWs

**Figure 2:**
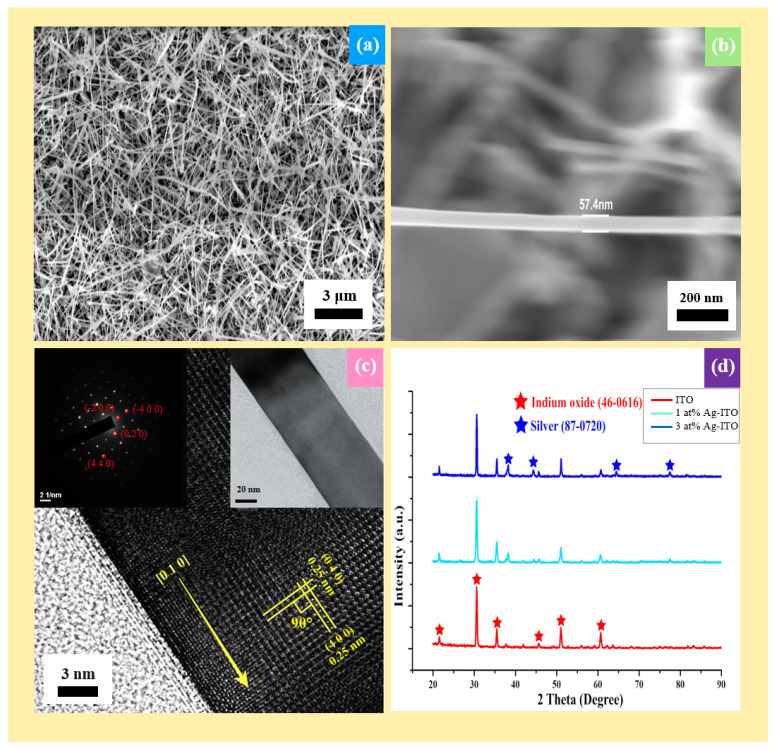
Structural characterization of ITO NWs (**a**) SEM image of ITO NWs (**b**) SEM image of a cross-section of an ITO NW (**c**) HR-TEM image of the ITO NW, showing two interplanar spacings of 0.25 nm corresponding to (0 4 0) and (4 0 0) planes. Insets are the SAED pattern and low-magnification TEM image of the NW. (**d**) XRD spectra, where the red line denotes ITO, the light blue line denotes 1 at% Ag-ITO NW, and the blue line denotes 3 at% Ag-ITO NW.

**Figure 3:**
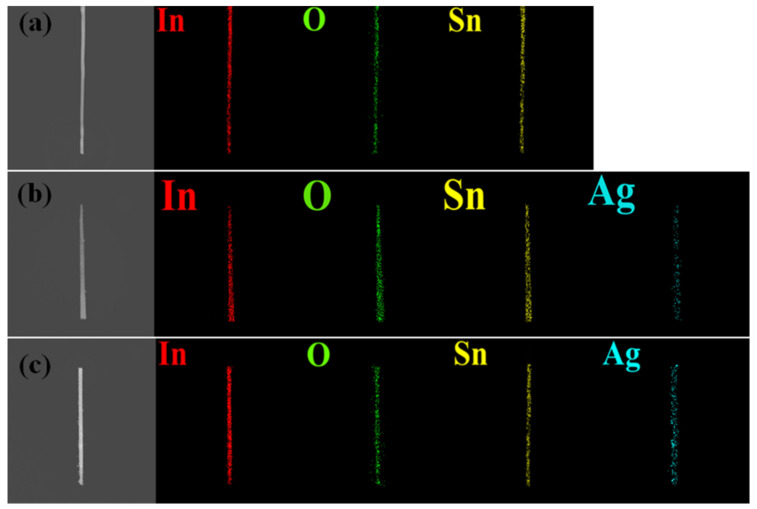
EDS mapping of the (**a**) ITO NW (**b**) 1 at% Ag-ITO NW (**c**) 3 at% Ag-ITO NW.

As-synthesized ITO NWs exhibited a high density and homogeneous morphology with a length of 5~10 μm and rectangular cross-section, as shown in Figure 2a,b. In addition, the average diameter of the ITO NWs is about 58.1 nm, typically (see Figure S1). Figure 2c is the HR-TEM image of an ITO NW, where it can be found that the growth direction of the ITO NW is [0 1 0]; both the interplanar spacings of (0 4 0) and (4 0 0) are 0.250 nm, and the angle between them is 90°. The upper-left and upper-right insets of Figure 2c are the selected area electron diffraction (SAED) pattern and a low-magnification TEM image, respectively. For further confirmation of the crystal structure, XRD analysis was performed, as shown in Figure 2d, where the red line at the bottom denotes the XRD signal of the ITO nanowire. The strongest peaks of the signal are located at 30.6° (2 2 2), 35.4° (4 0 0), and 51.0° (4 4 0), corresponding to JCPDS card no. 06-0416 (peaks marked with red stars). The brighter diffraction points (4 0 0) and (4 4 0) in the SAED pattern also correspond to the main peaks in XRD, demonstrating that the main structure of the ITO NW is indium oxide. The nanowire has been confirmed to be indium oxide of the bixbyite structure through TEM and XRD studies. The internal lattice of the nanowire is complete and the half-width ratio of the XRD peaks are all very small, indicating that the crystallinity of the grown ITO NW is high. The crystal lattice on the surface of the nanowire is different from the interior, as shown in the HR-TEM image in Figure 2c; the lattice structure of the interior of the nanowire is complete, while the lattice of the margin is incomplete. It is inferred that the ITO NWs prepared in the experiment were not annealed after the reaction, which resulted in a large number of oxygen vacancies generated on the surface of the nanowire; thus, there could be many oxygen vacancies on the surface [26], which would be verified by XPS and PL in this work. From the analyses of TEM and XRD, it is difficult to see the presence of tin, since the amount of doped tin atoms is few and the atomic radius of tin is similar to that of indium. Therefore, tin oxide hardly affects the lattice structure of indium oxide, the main component of the nanowire. In order to confirm whether the tin atoms were successfully doped and to understand the composition of the nanowire, elemental analysis was performed by EDS elemental mapping, as shown in Figure 3a, indicating that tin was successfully doped and evenly distributed throughout the entire nanowire. The amount of tin was 3% of the atomic ratio in the single NW. Based on a previous report [27], the tin in ITO was lower than 10% and there were almost no obvious peaks under XRD, which is coherent with our XRD results.

**Figure 4:**
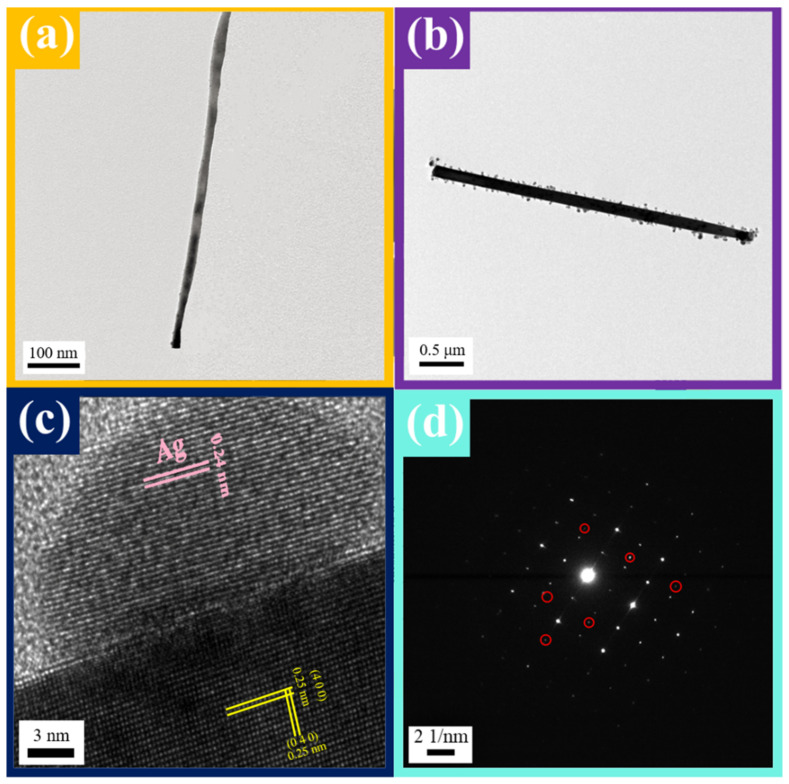
The low-magnification TEM images of (**a**) a 1 at% Ag-ITO NW (**b**) a 3 at% Ag-ITO NW (**c**) HR-TEM image of the Ag-ITO NW showing the lattice of Ag nanoparticle; (**d**) SAED pattern of the Ag-ITO NW showing the new diffraction spots in the ITO NW.

From TEM images in Figure 4a,b, it can be observed that many nanoparticles were deposited on the surface of the nanowire, and the number of Ag nanoparticles of 3 at% Ag-ITO NW is significantly more than that of 1 at% Ag-ITO NW. Moreso, the diameter of Ag nanoparticles of 3 at% Ag-ITO, which is about 3~5 nm, is larger than that of 1 at% Ag-ITO, which is about 1~3 nm. Figure 4c is the HRTEM image of Ag-ITO, where the interplanar spacing of the Ag particle is 0.24 nm, corresponding to (111) of Ag. Additionally, there are some extra points in the SAED pattern of the Ag-ITO NW, as shown in Figure 4d, while they do not appear in the SAED pattern of ITO NW. XRD was used for identification; Figure 2d shows XRD analysis, where the light blue line denotes 1 at% Ag-ITO, and the blue line denotes 3 at% Ag-ITO, respectively. There are four extra peaks at 38.2° (1 1 1), 44.4° (2 0 0), 64.6° (4 2 0), and 77.9° (2 2 2), corresponding to JCPDS card no. 87-0720 (marked with blue stars), and the signal of 3 at% Ag-ITO NW has stronger intensity. Before and after Ag modification, the nanowire lattice did not change, as shown in the HRTEM images of Figure 2c and Figure 4c. Thereby, it can be concluded that Ag nanoparticles existed on the surface of the nanowire rather than inside the nanowire. The Ag could not be quantified by EDS since the amount of the decorated Ag was too little and the Ag was in the form of particles uniformly distributed on the surface. Therefore, we used EDS elemental mapping to quantify the proportion of the decorated Ag atoms. Figure 3b is the mapping image of 1 at% Ag-ITO NW, while Figure 3c is the mapping image of 3 at% Ag-ITO NW. There are many Ag signals (in light blue) on the surface of NW, and the Ag signal of 3 at% Ag-ITO NW is more than that of 1 at% ITO NW, demonstrating the existence and quantity of silver.

**Figure 5:**
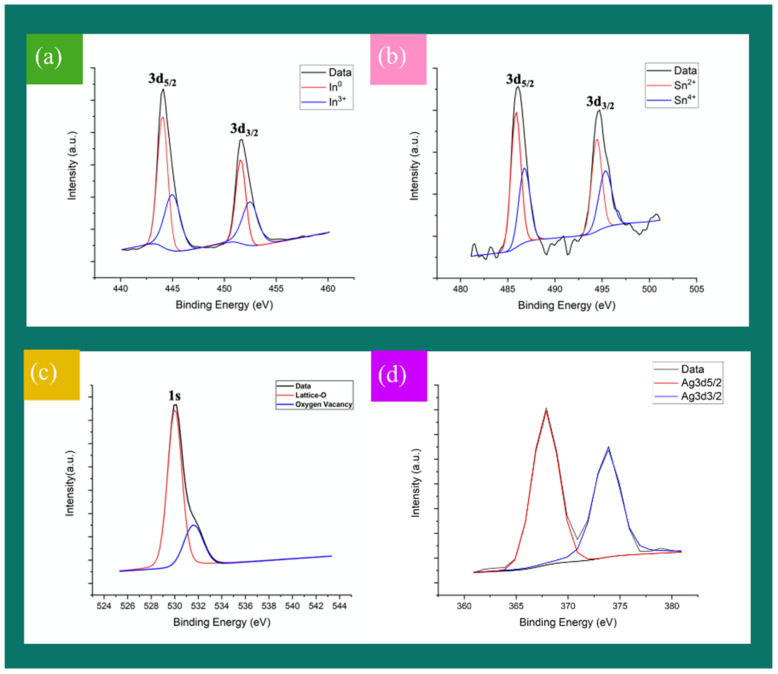
XPS interpretation of spectra for (**a**) In (**b**) Sn (**c**) O (**d**) Ag.

Next, we further identified the composition of the nanowires by XPS analysis. Figure 5 is the XPS interpretation of the spectra for the ITO and Ag-ITO NWs, and three of the main peaks are pulled out for peak separation. The two characteristic peaks of 3d_5/2_ and 3d_3/2_ orbits of In are composed of the peaks of In^0^ and In^3+^, as shown in Figure 5a; the bond energies are approximately 444.0 eV and 451.7 eV, respectively. Figure 5b shows the two characteristic peaks of 3d_5/2_ and 3d_3/2_ orbits of Sn and are composed of the peaks of Sn^2+^ and Sn^4+^; the bond energies are about 486.1 eV and 494.7 eV, respectively. Figure 5c shows the bond energy of 1s and orbital of oxygen is around 530 eV ± 0.1, which is consistent with the In-O bond energy. Furthermore, there is a peak at 531.7 eV due to the presence of oxygen vacancies, demonstrating that the surface lattice on the TEM image is incomplete. We found two more characteristic peaks at 367.6 eV and 373.6 eV, as shown in Figure 5d, corresponding to the bond energies of 3d_5/2_ and 3d_5/2_ orbits of silver, confirming successful modification of Ag atoms on the surface of the ITO NWs.

### 3.2. Photoluminescence Studies of ITO and Ag-ITO NWs

**Figure 6:**
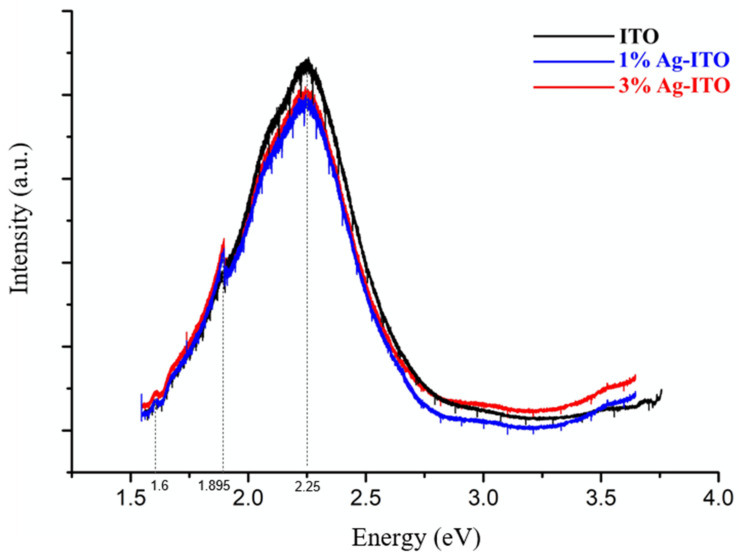
The room temperature PL analysis of the ITO NW in black, the 1 at% Ag-ITO NW in blue and the 3 at% Ag-ITO NW in red.

Generally, bulk ITO materials have a wide band gap, thus, they almost have no photoluminescent properties. However, due to the smaller band gap of ITO materials at the nanoscale, intense visible light emission can be observed. Figure 6 reveals the room temperature PL spectra of ITO and Ag-ITO NWs. The black peaks represent ITO nanowires. A small amount of indium oxide doped with tin does not cause much difference in luminescence properties [27]. There is a broad and strong peak at 2.25 eV, where there are many peaks superimposed. The peak at around 2.1 eV is related to the energy level difference from the lowest energy oxygen vacancy (V_0_) to the second-lowest energy indium vacancy (In^0^) [28]. The peak at around 2.2 eV is a green, fluorescent signal from the conduction band (CB) to V^+^_0_ [29], which is commonly investigated in the many PL spectra of products synthesized via the VLS route. Hence, the rest of the luminescence peaks related to oxygen vacancies were mentioned in previous studies around 2.45 eV and 2.69 eV [28], however, they are less obvious than the first two peaks. We presume the broad peak is formed by the superposition of four peaks together, mainly resulting from the first two peaks, thereby, the half-width of this peak is very large. There is a small peak at about 1.6 eV, which is related to the PL signal of the silicon substrate, while the peak at 3.5~3.6 eV is related to the phenomenon of the near-band-edge (NBE) emission [30]. The oxygen vacancies have a large specific surface area on the surface of the nanowire; thus, the intensity of visible light emission is much higher than that caused by the energy level of ITO itself. After the modification with silver, a small peak will appear at the position of about 1.895 eV, which is relevant to Ag nanoparticles [31], causing a low-energy luminescence emission due to additional energy bands among the ITO band gaps. For ITO thin films, there are four peaks at 2.26 eV, 2.66 eV, 2.84 eV, and 3.1 eV. The first three are attributed to transitions of V_0_^+^ to the valence band, V_0_^+^ to (V_In_–V_0_) hole, and V_0_^++^ to (V_In_–V_0_), respectively; the strongest intensity peak at 2.84 eV is a red fluorescent signal [32], while our PL analysis shows a green fluorescent signal for ITO nanowires.

### 3.3. Growth Mechanisms of ITO and Ag-ITO NWs

**Figure 7:**
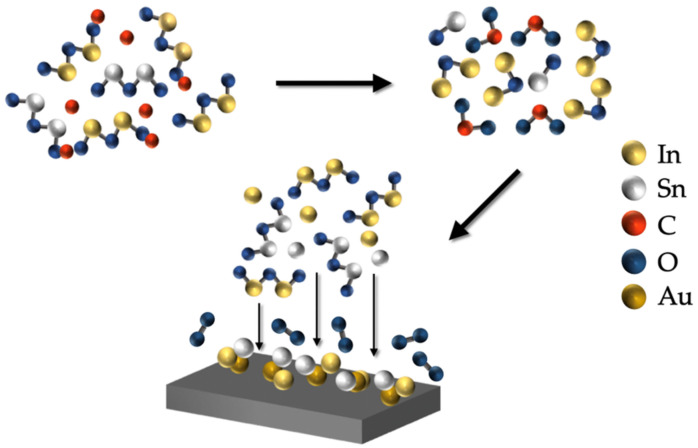
Schematic illustration of the growth mechanism of ITO NW, where the reaction follows the VLS route.

Figure 7 is the schematic illustration showing the growth mechanism of ITO NW. Both In_2_O_3_ and SnO_2_ are of high melting point; thereby, the carbothermic reduction method was used to make In_2_O_3_ and SnO_2_ unstable and evaporated. The growth mechanism of indium oxide and tin oxide has been studied [33,34]. First, the indium oxide and tin oxide were reduced to unstable intermediate In_2_O and SnO vapor through active charcoal. Here are the possible reactions at the front end of the tube furnace tube:

In_2_O_3(s)_ + C_(s)_ → In_2_O_(v)_ + CO_2(v)_ (1)

SnO_2(s)_ + C_(s)_ → 2SnO_(v)_ + CO_2(v)_ (2)

Then, the In_2_O and SnO vapor were transported at low-temperature downstream by the carrier gas and became more stable liquid In and Sn, which reacted with the Au catalyst on the substrate and formed an In-Au-Sn liquid alloy. As the reaction continued, the indium and tin in the droplet gradually reached saturation; when supersaturation was reached, indium and tin precipitated from the droplet and were oxidized into indium oxide and tin oxide until ITO nanowires were entirely formed. The possible reactions downstream of the tube furnace are:

3In_2_O_(v)_ → 4In_(l)_ + In_2_O_3(s)_ (3)

In_(l)_ + O_2(v)_ → In_2_O_3(s)_ (4)

SnO_(v)_ → 2Sn_(l)_ + SnO_2(s)_ (5)

Sn_(l)_ + O_2(v)_ → SnO_2(s)_ (6)

Figure S3 is the low-magnification TEM image of a single nanowire showing the Au catalyst at the top of a nanowire, demonstrating that nanowires were synthesized via the VLS route.

In the electrochemical system, AgNO_3_ and KNO_3_ were dissociated to Ag^+^, K^+^, NO_3_^−^ ions. With the applied voltage, Pt (CE) transferred electrons to the cathode (WE); thus, Ag^+^ ions were attracted by WE through a negative charge. Furthermore, Ag^+^ ions were reduced to Ag atoms. Here is the reaction:

Ag^+^ + e^−^ → Ag_(s)_ (7)

### 3.4. Electrical Measurements of Single ITO NW and Ag-ITO NW

**Figure 8:**
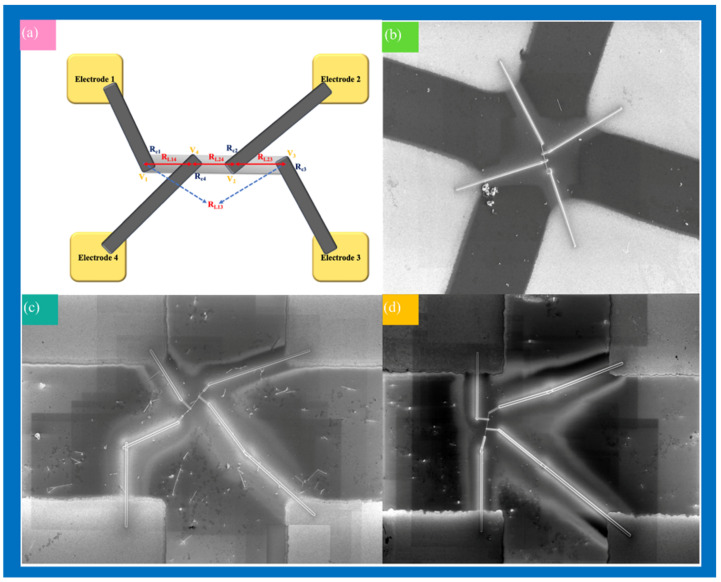
(**a**) Schematic illustration of the electrical measurement setup. SEM images of the measured (**b**) ITO NW, (**c**) 1 at% Ag-ITO NW, (**d**) 3 at% Ag-ITO NW, connected to four metal pads.

Figure 8 is the electrical resistance measurements of the single ITO, 1 at% Ag-ITO, and 3 at% Ag-ITO NW with the method introduced by Gu et al., as previously mentioned. The schematic illustration of the electrical measurement setup is shown in Figure 8a, while Figure 8b–d are SEM images of a single ITO NW 1 at% Ag-ITO NW, and 3 at% Ag-ITO NW connected to electrodes, respectively. The resistance between different electrodes was measured with the two-contact method. We measured a single nanowire 8 times, including R_13_, R_14_, R_23_, R_24_ with an applied positive and negative voltage. The measurements of single ITO, 1 at% Ag-ITO, and 3 at% Ag-ITO NW are shown in Figures S4–S6, respectively. Through the appropriate calculations, the resistivity of a single nanowire was obtained without errors from contact resistances and the Schottky voltage drop, as shown in Table S1, where the resistivity of a single ITO NW was 4.02 × 10^−6^ Ω·m, that of a single 1 at% Ag-ITO NW was 9.85 × 10^−5^ Ω·m, and that of a single 3 at% Ag-ITO NW was 3.55 × 10^−4^ Ω·m. Moreso, the resistivity standard deviation of a single ITO NW was 6.82 × 10^−5^ Ω·m, that of a single 1 at% Ag-ITO NW was 9.85 × 10^−5^ Ω·m, and that of a single 3 at% Ag-ITO NW was 2.4 × 10^−4^ Ω·m. The lower resistivity of ITO NW may be attributed to a higher concentration of oxygen vacancies on the surface of the nanowire and little Sn doping, making the nanowire more conductive [35,36]. Based on the results, the resistivity increased with the increasing surface modification of Ag. Since the heterojunction was formed between Ag and ITO NW, the electrons on the ITO conduction band were captured by Ag and the energy band near the surface was more curved, resulting in a larger electron depletion layer [37]. In view of the space of the electron conduction channel, when electrons are transferred, a larger energy barrier is encountered; thus, the resistivity is higher than compared to pure ITO NW. The great conductivity of ITO NWs makes them an attractive choice for interconnection in nanodevices. Additionally, with heterojunction formed, Ag-decorated ITO NWs may be promising materials for gas sensors. Table S1 summarizes the gas sensing studies on the heterojunctions based on ITO NW, thin films and silver nanoparticle modified nanowire [38,39,40]. The resistivity we measured for an ITO nanowire is close to the previously reported resistivities of ITO thin films [41,42,43].

## 4. Conclusions

We report the synthesis of ITO nanowires modified with Ag nanoparticles through two steps. In the first step, high-density ITO NWs were fabricated with chemical vapor deposition by the VLS route. The growth mechanism of the ITO NWs has been proposed. We conducted the composition and structure identification of the ITO NWs with TEM, EDS, XRD and XPS, respectively. The ITO nanowires were found to have visible light-emitting properties and numerous oxygen vacancies on the surface, based on PL analyses. In the second stage, we utilized an electrochemical method to decorate the ITO NWs with 1 at% and 3 at% Ag nanoparticles. HRTEM and EDS studies confirmed that the Ag nanoparticles were distributed on the surface of the ITO NWs, rather than in the interior. PL studies show that an extra Ag signal at around 1.895 eV appeared for the Ag-ITO NWs. The single ITO nanowire was of a very low resistivity; resistivity increased with more surface modification of Ag, attributed to the change of the electron transmission channel of the Ag-ITO NW. This study supports the development of future applications of ITO NWs on electronic and optoelectronic devices, including solar cells, supercapacitors and flexible LEDs.

## Supplementary Materials

The following supporting information can be downloaded at https://www.mdpi.com/article/10.3390/nano12060897/s1, Figure S1: Gaussian distribution and corresponding histogram of the transverse dimensions of ITO NWs, Figure S2: Schematic illustration of fabrication process of electrical measurement nanodevice, Figure S3: TEM image showing the Au catalyst on top of an ITO NW, Figure S4: I-V measurements of ITO NW (a) R13+ (b) R13− (c) R14+ (d) R14− (e) R23+ (f) R23− (g) R24+ (h) R24−. Figure S5: I-V measurements of 1 at% ITO NW (a) R13+ (b) R13− (c) R14+ (d) R14− (e) R23+ (f) R23− (g) R24+ (h) R24−, Figure S6: I-V measurements of 3 at% ITO NW (a) R13+ (b) R13− (c) R14+ (d) R14− (e) R23+ (f) R23− (g) R24+ (h) R24−, Table S1: Previous studies on ITO heterojunction and silver nanoparticle decorated nanowire.
