# Supplementary material for: Synthesis and Characterization of Indium Tin Oxide Nanowires with Surface Modification of Silver Nanoparticles by Electrochemical Method
Source: Nanomaterials (Basel). 2022 Mar 8;12(6):897. doi: 10.3390/nano12060897 (PMC8950758; doi:10.3390/nano12060897)
Supplement: Supplementary file 1 [file nanomaterials-12-00897-s001.zip › nanomaterials-1595320-supplementary.pdf]

nanomaterials

Supporting Information

Synthesis and Characterization of Indium Tin Oxide Nanowires with Surface Modification of Silver Nanoparticles by Electrochemical Method

Shu-Meng Yang [1], Hsi-Kai Yen [1] and Kuo-Chang Lu [1,2,*]

[1] Department of Materials Science and Engineering, National Cheng Kung University, Tainan 701, Taiwan; n56074287@gs.ncku.edu.tw (S.-M.Y.); n56091302@gs.ncku.edu.tw (H.-K.Y.)
[2] Core Facility Center, National Cheng Kung University, Tainan 701, Taiwan
* Correspondence: gkclu@mail.ncku.edu.tw; Tel.: +886-6-275-7575 ext.62920

List of contents

Figure S1 | Gaussian distribution and corresponding histogram of the transverse dimensions of ITO NWs.
Figure S2 | Schematic illustration of fabrication process of electrical measurement nanodevice.
Figure S3 | TEM image showing the Au catalyst on top of an ITO NW.
Figure S4 | I-V measurements of ITO NW (a) R13+ (b) R13- (c) R14+ (d) R14- (e) R23+ (f) R23- (g) R24+ (h) R24-.
Figure S5 | I-V measurements of 1 at% ITO NW (a) R13+ (b) R13- (c) R14+ (d) R14- (e) R23+ (f) R23- (g) R24+ (h) R24-.
Figure S6 | I-V measurements of 3 at% ITO NW (a) R13+ (b) R13- (c) R14+ (d) R14- (e) R23+ (f) R23- (g) R24+ (h) R24-.
Table S1 | Previous studies on ITO heterojunction and silver nanoparticle decorated nanowire.

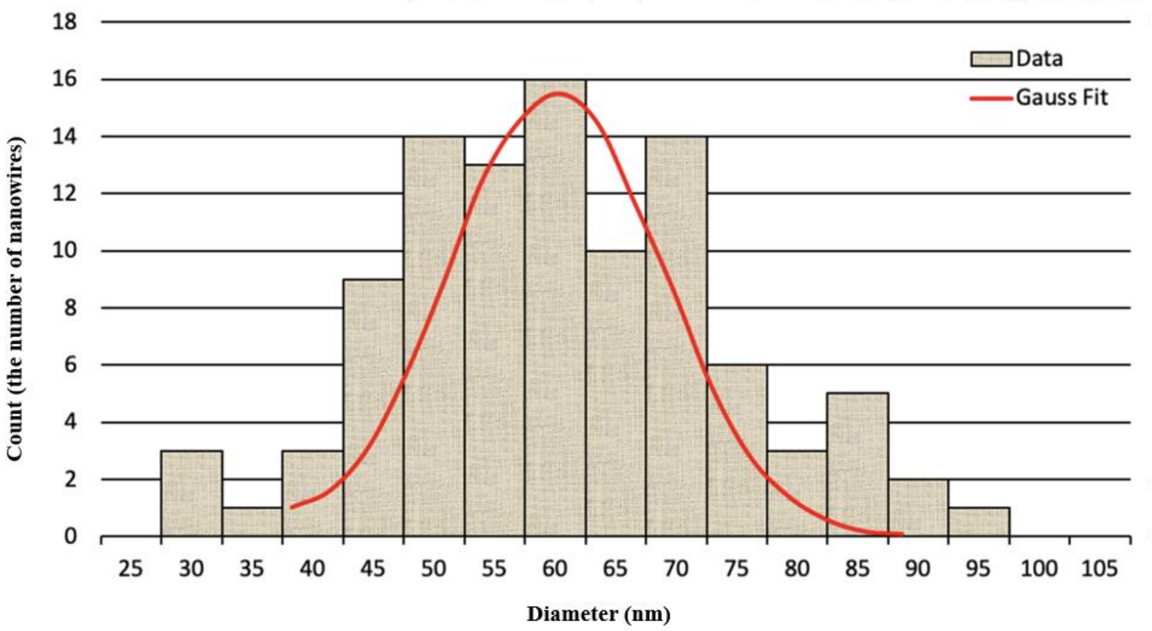

Figure S1. Gaussian distribution and corresponding histogram of the transverse dimensions of ITO NWs.

We selected independent 100 strips of ITO NWs and measured their diameter; Figure S1 shows the gaussian distribution and histogram based on the results. The diameter of ITO NWs are ranged between 45~75 nm. The average diameter is 58.1 nm with standard deviation of ±13.54 nm.

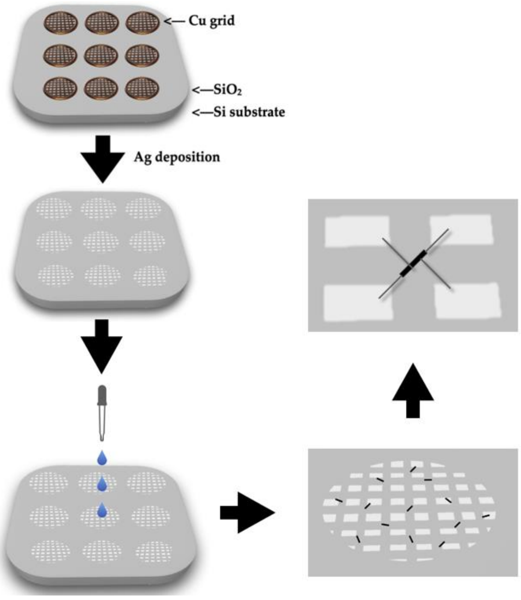

Figure S2. Schematic illustration of fabrication process of electrical measurement nanodevice.

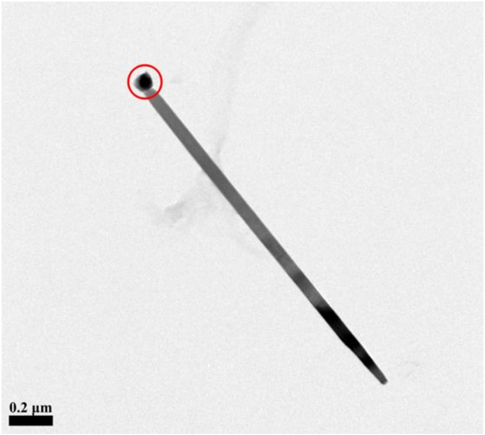

Figure S3. TEM image showing the Au catalyst on top of an ITO NW.

Figure S3 reveals the Au catalyst on top of an ITO nanowire, demonstrating the growth mechanism. Figure S4, S5 and S6 show the I-V measurements for ITO NW, 1 at% Ag-ITO NW and 3 at% Ag-ITO NW, respectively. The resistances of R_{13}, R_{14}, R_{23}, R_{24} were measured by applying voltage from 0 to 1 V and 0 to -1 V.

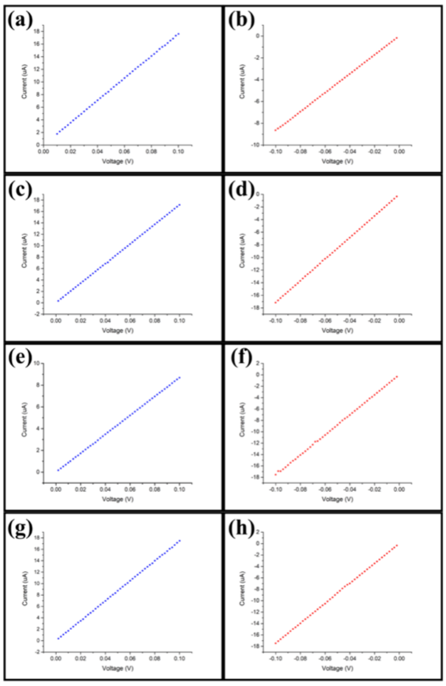

Figure S4. I-V measurements of ITO NW (a) R_{13+} (b) R_{13-} (c) R_{14+} (d) R_{14-} (e) R_{23+} (f) R_{23-} (g) R_{24+} (h) R_{24-}.

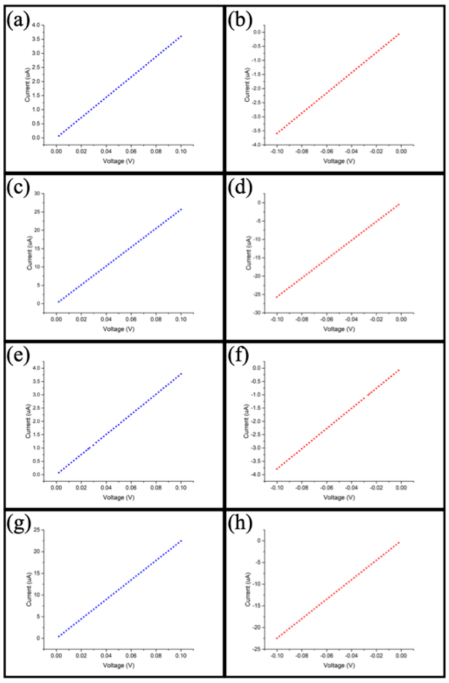

Figure S5. I-V measurements of 1 at% ITO NW (a) R_{13+} (b) R_{13-} (c) R_{14+} (d) R_{14-} (e) R_{23+} (f) R_{23-} (g) R_{24+} (h) R_{24-}.

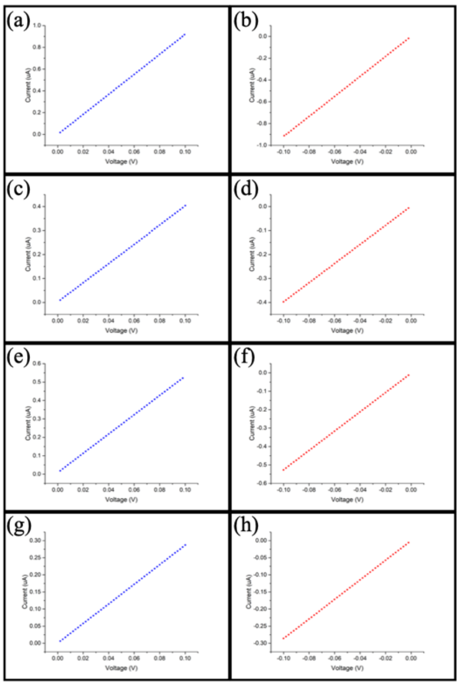

Figure S6. I-V measurements of 3 at% ITO NW (a) R_{13+} (b) R_{13-} (c) R_{14+} (d) R_{14-} (e) R_{23+} (f) R_{23-} (g) R_{24+} (h) R_{24-}.

Table S1. Previous studies on ITO heterojunction and silver nanoparticle decorated nanowire.

Structure	Gas type of gas sensing	Reference
SnO_2-ITO two-layer thin film	NO_2, H_2, NH_3, C_2H_5OH	[1]
Au-ITO thin film	NH_3	[2]
Ag-TiO_2 nanowire	NO_2	[3]

References

1. Yang, M.; Hong, S.H. Fabrication of ITO/SnO2 two-layer thin films and their gas sensing properties. J. Electrochem. Soc. 2010, 157, J392.
2. Hsu, C.S.; Chen, H.I.; Lin, C.W.; Chen, T.Y.; Huang, C.C.; Chou, P.C.; Liu, W.C. Ammonia gas sensing performance of an indium tin oxide (ITO) based device with an underlying Au-nanodot layer. J. Electrochem. Soc. 2012, 160, B17.
3. Abideen, Z.U.; Kim, J.H.; Kim, S.S. Optimization of metal nanoparticle amount on SnO2 nanowires to achieve superior gas sensing properties. Sens. Actuators B Chem. 2017, 238, 374–380.
